# P04.71. Acupuncture, self-care homeopathy, and practitioner-based homeopathy: comparing users in the 2007 NHIS

**DOI:** 10.1186/1472-6882-12-S1-P341

**Published:** 2012-06-12

**Authors:** J Frye

**Affiliations:** 1 Center for Integrative Medicine, Univ. of Maryland School of Medicine, Baltimore, USA

## Purpose

To compare non-users and users of acupuncture, homeopathy as self-care, and homeopathy with a practitioner. The 2002 and 2007 NHIS CAM Supplements indicate more users of homeopathy than of acupuncture; however, there were more visits to acupuncture practitioners suggesting that a large component of homeopathic use is for self-care.

## Methods

Data from the 2007 NHIS, including 23,393 adults, were used to compare recent users of acupuncture, homeopathy as self-care, homeopathy with a practitioner, and non-users regarding demographics, health behaviours, reasons for use, and perceived health.

## Results

All users were significantly more likely than the general population to be female, to have a Bachelor’s or higher education and to describe their health as good to excellent. However, acupuncture users were significantly more likely to score in the range of Serious Mental Illness on the K6 depression scale. Self-care homeopathy was most used by individuals of multiple race (4.4%), followed by Native Americans (3.8%); practitioner guided homeopathy by Asian Indians (2.1%); and acupuncture by Chinese (6.8%). Whites seemed more likely to use homeopathy (1.9%) than acupuncture (1.6%), but the difference was not significant (p=0.10). Of the conditions listed, homeopathy was most used for respiratory infections and acupuncture for back pain. All users were significantly more likely than the general population to have delayed or not obtained medical care because of cost. In contrast, they were also more likely to have income from interest and investments. Realistic estimates of actual expenditures are difficult due to the bimodal nature of the question asked with a threshold of $500.

## Conclusion

The relationship between acupuncture use and depression deserves further investigation. Given high levels of concern about overuse of antibiotics in respiratory infections, further research into the efficacy and cost-effectiveness of homeopathy for these conditions is warranted. Hopefully, future versions of NHIS-CAM will provide more realistic estimates of expenditures.

